# Somatic and sociodemographic predictors of depression outcome among depressed patients with coronary artery disease - a secondary analysis of the SPIRR-CAD study

**DOI:** 10.1186/s12888-019-2026-6

**Published:** 2019-02-04

**Authors:** Frank Vitinius, Steffen Escherich, Hans-Christian Deter, Martin Hellmich, Jana Jünger, Katja Petrowski, Karl-Heinz Ladwig, Frank Lambertus, Matthias Michal, Cora Weber, Martina de Zwaan, Christoph Herrmann-Lingen, Joram Ronel, Christian Albus

**Affiliations:** 1 Department of Psychosomatics and Psychotherapy, University of Cologne, Cologne, Germany; 2 Department of Psychiatry and Psychotherapy, University of Cologne, Cologne, Germany; 3 Department of Psychosomatics and Psychotherapy, Charité Universitaetsmedizin Berlin, Campus Benjamin Franklin, Berlin, Germany; 4 Institute of Medical Statistics and Computational Biology (IMSB), University of Cologne, Cologne, Germany; 5 German National Institute for state examinations in Medicine, Pharmacy and Psychotherapy, Mainz, Germany; 6 Department of Psychotherapy and Psychosomatic Medicine, Technical University Dresden, Dresden, Germany; 7 German Research Center of Environmental Health, Helmholtz Zentrum Muenchen, Institute of Epidemiology, Oberschleißheim, Germany; 8 Department of Psychosomatic Medicine and Psychotherapy, University Hospital of Mainz, Mainz, Germany; 9 Department of Psychosomatic Medicine and Psychotherapy, Hannover Medical School, Hannover, Germany; 10 Department of Psychosomatic Medicine and Psychotherapy, University of Goettingen Medical Center and German Center for Cardiovascular Research (DZHK), Partner Site Goettingen, Goettingen, Germany; 11 Department of Psychosomatic Medicine and Psychotherapy, University Hospital Rechts der Isar, Technische Universitaet München, Munich, Germany

**Keywords:** Depression - mental disorders - coronary heart disease - psychotherapy - type D personality

## Abstract

**Background:**

Depressive symptoms are common in patients with *coronary artery disease* (CAD) and are associated with an unfavourable outcome. Establishing prognostic patient profiles prior to the beginning of mental health care may facilitate higher efficacy of targeted interventions. The aim of the current study was to identify sociodemographic and somatic predictors of depression outcome among depressed patients with CAD.

**Methods:**

Based on the dataset of the multicentre SPIRR-CAD randomised controlled trial (*n* = 570 patients with CAD and ≥ 8 points on the *Hospital Anxiety and Depression Scale* (HADS)), 141 potential sociodemographic and somatic predictors of the change in the HADS-D depression score from baseline to 18-month-follow-up were derived in two different ways. We screened for univariable association with response, using either analysis of (co)variance or logistic regression, respectively, both adjusted for baseline HADS-D value and treatment group. To guard against overfitting, multivariable association was evaluated by a linear or binomial (generalised) linear model with lasso regularisation, a machine learning approach. Outcome measures were the change in continuous HADS-D depression scores, as well as three established binary criteria. The *Charlson Comorbidity Index* (CCI) was calculated to assess possible influences of comorbidities on our results and was also entered in our machine learning approach.

**Results:**

Higher age (*p* = 0.002), unknown previous myocardial infarction (*p* = 0.013), and a higher heart rate variability during numeracy tests (*p* = .020) were univariably associated with a favourable depression outcome, whereas hyperuricemia (*p* ≤ 0.003), higher triglycerides (*p* = 0.014), NYHA class III (*p* ≤ 0.028), state after resuscitation (*p* ≤ 0.042), intake of thyroid hormones (*p* = 0.007), antidiabetic drugs (*p* = 0.015), analgesic drugs (*p* = 0.027), beta blockers (*p* = 0.035), uric acid drugs (*p* ≤ 0.039), and anticholinergic drugs (*p* = 0.045) were associated with an adverse effect on the HADS-D depression score. In all analyses, no significant differences between study arms could be found and physical comorbidities also had no significant influence on our results.

**Conclusion:**

Our findings may contribute to identification of somatic and sociodemographic predictors of depression outcome in patients with CAD. The unexpected effects of specific medication require further clarification and further research is needed to establish a causal association between depression outcome and our predictors.

**Trial registration:**

www.clinicaltrials.gov NCT00705965 (registered 27th of June, 2008).

www.isrctn.com ISRCTN76240576 (registered 27th of March, 2008).

**Electronic supplementary material:**

The online version of this article (10.1186/s12888-019-2026-6) contains supplementary material, which is available to authorized users.

## Background

Depressive symptoms are common in patients with *coronary artery disease* (CAD) [1–3] with an estimated prevalence of about 20% [1, 4]. Recent meta-analyses showed an increased risk of mortality and new cardiovascular events in CAD patients with comorbid depression [5–7]. One meta-analysis found that post-myocardial infarction depression is associated with a 2.7-fold risk of impaired cardiovascular outcome and prognosis, independent from other risk factors [8].

Besides the influential aspects of depression on CAD, the involvement of depression in the etiology of CAD has been discussed, with an adjusted effect estimate of 1.6–1.9 for CAD onset among depressed patients [6]. Due to the great importance of depression as a prognostic factor in CAD with impaired cardiovascular outcome, Lichtman et al. (2014) claim that depression should be elevated to the status of a risk factor for adverse medical outcome in patients with acute coronary syndrome [5].

The pathophysiological mechanisms underlying the association between depression and heart disease have not been proven, but there is evidence that platelet hyperreactivity and coagulation, endothelial dysfunction, inflammatory activation, autonomic and neuroendocrine dysregulation in depression might mediate the increased risk [9–12]. An explanation of the link between depression and CAD is most likely multifactorial [13]. Sociodemographic and psychosocial factors also contribute to depression and cardiovascular outcome [1].

Research has been done on the prediction of depression outcome among heterogeneous patient samples with various treatments and outcome variables. Apart from the above-mentioned sociodemographic variables, there are also somatic variables that predict treatment benefit, for example blood biomarkers such as the “brain-derived neurotrophic factor” [14, 15], inflammatory markers [16, 17], brain activation patterns observed by imaging studies [18, 19] or EEG biomarkers such as quantitative EEG measures, connectivity measures, and event-related potentials [20–22]. Many of the predictors of poor response to antidepressive treatment are at the same time cardiac risk markers, for example elevated inflammatory markers, physical inactivity or thyroid dysfunction [23, 24]. Recent reviews addressing the association of depression and CAD were limited by a lack of data on the course of depression. They mostly presume an association between specific biological or sociodemographic variables and depression at baseline, but the response to antidepressive treatment in patients with comorbid CAD has been less intensively studied.

Establishing physiological and socio-demographic variables that contribute to depression outcome among patients with CAD and thus identifying subgroups of depressed CAD patients may benefit the treatment of depression, as well as the treatment of CAD and facilitate mental health support [25]. There is substantial interest in improving clinical outcome in patients with CAD, regarding the treatment of comorbid depression and observing its possible etiological and prognostic effects on CAD.

The main aim of the multicentre randomised controlled *Stepwise Psychotherapy Intervention for Reducing Risk in Coronary Artery Disease* (SPIRR-CAD) trial was to evaluate the effects of a stepwise combined short-term psychodynamic and cognitive-behavioral psychotherapy intervention on depressive symptoms in 570 patients with CAD and depression-scores ≥8 on the *Hospital Anxiety and Depression Scale* (HADS-D), compared to enhanced *treatment as usual* (TAU) [26].

In the trial, both groups showed a decrease of depression scores but improvement in the intervention group was not superior to that found with treatment as usual. However, the intervention tended to be beneficial for depressed CAD patients with Type D personality.

Based on the dataset of the SPIRR-CAD trial [26, 27], the aim of this paper was to determine potential somatic and socio-demographic baseline predictors of depression outcome, both overall and separately for the intervention and control group and to investigate their influence on the change of depressive symptoms between baseline and 18 months. Our exploratory approach may identify further variables contributing to depression outcome, such as comorbid or other somatic conditions.

## Methods

### Study organisation, participants and treatment

Patients for the SPIRR-CAD trial were recruited and treated at ten different study sites across Germany (see [27] for further information). 570 patients with any manifestation of CAD (from stable angina to acute coronary syndrome), recent coronary angiograms and depression-scores ≥8 on the *Hospital Anxiety and Depression Scale* (HADS-D) were randomised into two parallel arms (T0b). Exclusion criteria were severe heart failure (left ventricular ejection fraction < 20% or NYHA class IV), life-threatening conditions, severe inflammatory disease, severe depressive episodes according to SCID diagnoses, severe mental illnesses (e.g. bipolar affective disorder, psychotic disorder, dementia, persistent drug abuse) and acute suicidal tendencies. The intervention group received three sessions of individual, supportive-expressive psychotherapy. Four to eight weeks later (T1), patients were re-evaluated and only those with persisting symptoms of depression (T2), were offered an additional 25 sessions of group psychotherapy over ten months. Patients in the control group received one 30-min tailored education session on health behaviour and psychosocial aspects of CAD. Screening for depressive symptoms was made at T0a (median 2–3 weeks before randomisation), while more detailed diagnostic assessments were made at the baseline visit immediately before randomisation (T0b), after 4–8 weeks (T1), after 6 months (T2), 12 months (T2b), 18 months (T3) and 24 months (T4). Further information on the study organisation, sample recruitment, randomisation and treatment can be found elsewhere [26, 27].

### Outcomes

As in the SPIRR-CAD [26] randomised controlled trial, our defined endpoint was the change in HADS-D depression scores from baseline to 18 months (T3 minus T0b), which corresponded to the end of group treatment. We supplemented our analyses, using three established binary criteria for depression. Criterion A was defined as a 50% improvement in HADS-D depression score from T0b to T3, criterion B was defined as a HADS-D depression score lower than eight at T3, whereas criterion C represented an improvement of more than four points in the HADS-D depression score.

### Materials

The dataset of the SPIRR-CAD randomised controlled trial comprised, among others, data on medical history (e.g. myocardial infarction, bypass surgery, number of events, etc.), blood tests, current drug use and socio-demographic data (e.g. education, monthly income, living with a partner, etc.). A list of all 141 screening variables can be found in an additional file [see Additional file 1, “Screening variables of the SPIRR-CAD dataset”].

### Charlson comorbidity index

The *Charlson Comorbidity Index* (CCI) [28, 29] is a well validated instrument to assess the degree and burden of somatic comorbidities. It was originally designed to predict one-year mortality in patients with several comorbidities. The evaluation consists of 17 possible comorbidities that are valued from one to six points (range 0–30).

### Statistical analyses

Empirical distributions of qualitative (categorical) variables were summarized by count (percentage), those of quantitative variables by either mean ± standard deviation (SD) or median (25th to 75th percentile), depending on distributional characteristics (i.e. apparent skewness). “Univariable” associations (i.e. *p* < 0.05) with depression outcome measures were evaluated by means of analysis of covariance and logistic regression, respectively (with assigned treatment and baseline HADS-D value as covariables). To guard against overfitting, multivariable association of all 141 variables was evaluated by a linear or binomial (generalised) linear model with lasso regularisation (R package glmnet 2.0–16, a machine learning approach (see also [30]). The Charlson Comorbidity Index was also entered in the machine learning approach, representing one of the 141 variables. Beforehand, 5 datasets were completed by multiple imputation based on an iterative Markov Chain Monte Carlo method with full conditional specification and predictive mean matching. For subsequent analyses each observation was assigned a weight of 1/5. The penalty parameter lambda determining feature selection was chosen by 10-fold cross-validation (CV) to minimize mean-squared error or model deviance, respectively. Shrunken coefficients (B) or anti-logs of these (Exp(B), corresponding to odds ratios) are presented with 95% confidence intervals and log(lambda) values, the latter to describe the order of importance (“ranking”) for prediction. Only the first 10 selected features (out of more than 100 variables in total) per model are presented. The selection proportion for each feature and 95% confidence intervals for B and Exp(B) were estimated by 10,000 bootstrap replications (R package boot 1.3–20). Goodness-of-fit was characterized by mean-squared CV error and standard deviation or (predictive) odds ratio (pOR) with 95% confidence interval, respectively. Statistical calculations were done with SPSS Statistics 25 (IBM Corp., Armonk, NY, USA) and R 3.5.0 (R Foundation for Statistical Computing, Vienna, Austria).

## Results

Demographic and medical characteristics of the study cohort are shown in Table 1. Our model with lasso regularisation identified a variety of potential predictive variables of depression outcome [see Additional file 2: Table S2]. Since many coefficients (e.g. 135 for the continuous outcome measure) were non-zero, we focussed on the 10 first selected variables (i.e. with highest log(lambda) value). Since there was a big overlap with the solution of the “univariable” approach, any difference in coefficients may indicate the presence or amount of confounding or collinearity in the dataset.

**Table 1:** Baseline characteristics by study arm

	Intervention group (*n* = 285)		Control group (*n* = 285)	
Characteristics	*n*/total valid *n*	%	*n*/total valid *n*	%
Demographics				
Age, mean ± SD (total valid *n*), y	59.1 ± 9.8 (285)		59.3 ± 9.3 (285)	
Female sex	61/285	(21.4)	59/285	(20.7)
Married	170/268	(63.4)	185/271	(68.3)
Socioeconomic status				
Low	117/285	(45.0)	123/261	(47.1)
Medium	84/285	(32.3)	71/261	(27.2)
High	59/285	(22.7)	67/261	(25.7)
Baseline medical data				
Hypertension	252/281	(89.7)	245/280	(87.5)
Hyperlipidemia	236/273	(86.4)	240/270	(88.9)
Diabetes mellitus	69/275	(25.1)	70/279	(25.1)
TSH, mean ± SD (total valid *n*), mU/l	1.6 ± 1.1 (187)		1.5 ± 1.2 (175)	
BMI, mean ± SD (total valid *n*), kg/m^2^	28.5 ± 5.0 (280)		28.4 ± 4.8 (275)	
Smokers	90/282	(31.9)	97/284	(34.2)
Physically active	140/285	(49.1)	120/285	(42.1)
Previous myocardial infarction	139/271	(51.3)	161/273	(59.0)
Previous CABG	53/283	(18.7)	45/282	(16.0)
Recent acute myocardial infarction	93/285	(32.6)	94/285	(33.0)
Recent coronary intervention (PCI, CABG)	204/285	(71.6)	206/285	(72.3)
NYHA class I-II	240/285	(84.2)	242/285	(84.9)
NYHA class III	45/285	(15.8)	43/285	(15.1)
Charlson Comorbidity Index, median (IQR)	2	(1/3)	2	(1/3)
SF-36, mean ± SD (total valid *n*),	59.0 ± 24.9 (268)		55.6 ± 26.4 (271)	
Baseline medication				
Thyroid substitution	34/285	(11.9)	30/285	(10.5)
Continued intake (until T3)	11/285	(3.9)	14/285	(4.9)
Uric acid lowering agents	24/285	(8.4)	24/285	(8.4)
Antibiotics	2/285	(0.7)	2/285	(0.7)
ACE inhibitors	187/285	(65.6)	193/285	(67.7)
Aspirin	257/285	(90.2)	262/285	(91.9)
β-Blockers	246/285	(86.3)	257/285	(90.2)
Statins	256/285	(89.8)	265/285	(93.0)
Antidepressant medication	33/285	(11.6)	36/285	(12.6)
HADS-D depression subscale, mean ± SD (total valid *n*)				
Baseline	10.4 ± 2.5 (284)		10.4 ± 2.5 (284)	
After 18 months (T3)	8.7 ± 4.1 (284)		8.9 ± 3.9 (285)	

*TSH* Thyroid-stimulating hormone, *BMI* body mass index, *CABG* coronary artery bypass graft surgery, *PCI* percutaneous coronary intervention, *NYHA* New York Heart Association, *IQR* interquartile range, *SF-36* Short Form Health Survey – physical functioning-36-item, *ACE* Angiotensin-converting enzyme, *HADS-D* Hospital Anxiety and Depression Scale - German version (depression subscale)

Our continuous univariable model revealed a set of eight remaining variables significant at *p* ≤ .05. Four of them could also be found with the model of lasso regularisation, increasing the statistical power: age (*p* = .002), thyroid hormone substitution (*p* = .007), higher level of triglycerides (*p* = .014) and intake of analgesics (*p* = .027). Higher age was associated with a favourable outcome, older patients having a significantly more reduced HADS-D score at T3, whereas thyroid hormone substitution, a higher level of triglycerides, intake of analgesics and antibiotics were associated with a significantly smaller decrease in HADS-D score. The finding concerning antibiotics could not be considered as a clinically significant influence because of the small sample size of only 4 patients who received antibiotics. The influence of higher HADS-D baseline by itself on depression outcome could be shown in the model of lasso regularisation. The study arm had no significant influence on the outcome. The CCI had no significant influence on HADS-D depression score after 18 months (*p* = .238).

The consistent findings of both the model of criterion A (50% improvement from baseline to T3) and of the model with lasso regularisation were unfavourably related to hyperuricemia (*p* = .002), presence of uric acid lowering drugs (*p* = .039), NYHA class III (*p* = .028), and state after resuscitation (*p* = .042). Criterion B (HADS-D score < 8 at T3) also showed a significant, unfavourable influence of hyperuricemia (*p* = .001), condition after resuscitation (*p* < .001), NYHA class III (*p* < .001) and presence of uric acid drugs (*p* = .034). Presence of anticholinergic drugs was also associated with an adverse response after 18 months (*p* = .045), which is in line with our results of lasso regularisation.

Criterion C (> 4 points improvement at T3) and the model with lasso regularisation indicated a negative association with hyperuricemia (*p* = .003), presence of antidiabetic drugs (*p* = .015) and presence of beta blockers (*p* = .035). An unknown state after myocardial infarction (*p* = .013) and a higher heart rate variability during numeracy tests (*p* = .020) were associated with a better outcome. The variables “heart rate variability during dictated breathing rhythm” and “diameter of left atrium” were not interpretable because of too many missing values and an odds ratio of approximately 1, respectively.

Several variables showed significant results in the model of the main outcome criterion (HADS-D depression score T3 minus T0b) and the additional three criteria A, B and C. These variables are highlighted with different colours in Additional file 2: Table S2.

## Discussion

The purpose of this paper was to identify variables that could help to predict depression outcome and identify patients with CAD who show greater reductions of depressiveness after 18 months. Prior to this study, the extent to which somatic and socio-demographic variables predicted depression outcomes in patients with CAD was inconclusive. Several reviews assess the association between a specific biological or socio-demographic variable and depressive symptoms at one time-point, but the analysis of their influence on depression outcome is sparse. In addition, most of the literature deals with depressed patients in general and not the specific combination of depression and comorbid CAD. There is evidence that prior depression, cardiac history and Type D personality are risk factors for the persistence of depressive symptoms during the first year after myocardial infarction [31].

Our model revealed significant influence of patient’s age at baseline on depression change. Higher age was associated with a favourable response after 18 months. Previous literature on this topic deals with the efficacy of treatment in younger compared to older age groups but the comparison between these results is difficult. Study designs, interventions and study groups differ, as well as the number of therapy sessions or severity of depression at baseline. Few meta-analyses or reviews have taken these differences into account, in order to compare effect sizes of younger and older age groups. The authors did not find any significant difference in depression outcome between both groups [32, 33], but it is important to note that they did not analyse patients with comorbid CAD, specifically. There is evidence that higher age in coronary patients is a predictor for better scores in the *Mental Component Summary* (MCS), a summary of all mental dimensions of the SF-36 questionnaire [34, 35]. We have to consider that the older patients of the SPIRR-CAD dataset with comorbid CAD may differ from the younger patients, as mortality rates naturally increase with age. In addition, the older patients may have developed better coping strategies than the younger patients. Many factors that are associated with higher age and the number or severity of previous episodes of depression have to be considered [33, 36]. In our study we evaluated depressed patients with comorbid CAD, undergoing the previously described intervention therapy. This makes it even more difficult to compare our results with others. Although our data suggest a significant effect of patient’s age at baseline, the effect is small.

We detected an unexpected association between specific medications and depression outcome. Patients with thyroid hormone substitution at T0b were less likely to recover from depression. In contrast, literature shows that thyroid hormone intake may improve therapeutic response in antidepressive treatment [37–39], but it is important to note that none of these trials pay particular regards to comorbid CAD. A recent trial by Carney et al. (2016) found that high levels of the free thyroid hormone T4 predicted less improvement in depressive symptoms in patients with CAD [24]. Literature dealing with the association of thyroid hormone levels and depressive disorder differ in terms of analysed thyroid hormones (T3, T4, and TSH), depression rating scales, and patient characteristics (age, gender, comorbidities) with often small sample sizes. In addition, the mentioned literature evaluates treatment efficacy of depressive symptoms and is not comparable to our findings. We could only show the impact of thyroid hormones on depression outcome in general. One meta-analysis found that higher levels of the thyroid hormone thyroxine are associated with an increased risk of depression [40]. There is evidence that both hyper- and hypothyroidism are associated with changes in mood and intellectual performance [38, 40, 41] and it is well known that especially severe hypothyroidism can imitate depressive symptoms. Our dataset included only blood levels of thyroid stimulating hormone (TSH), but we could not find a significant association between hormone levels and changes in HADS-D scores. Moreover, this variable was only available in 362 of 570 subjects (63.5%) and further variables for assessing thyroid metabolism (T3, T4) are lacking. We do not know if our medicated patients suffer from clinical hypothyroidism or different conditions that require thyroid medication and the literature cannot be applied to our findings without restriction. A possible explanation of the negative association between depression recovery and thyroid medication intake could be a higher burden of somatic disease in patients with CAD, but it is important to note that the CCI could not show a significant influence on depressive symptoms after 18 months. There may also be possible drug interactions that cannot be detected in our study, influencing our results. Future studies should assess thyroid hormone levels (T3, T4, and TSH), as well as thyroid medication with regard to depression outcome, in order to ensure a clearer interpretation of the findings. The considerations above can also be applied to the unfavourable effect of uric acid drugs, analgesic drugs, anticholinergic drugs, antidiabetic drugs and beta blockers. There is evidence that beta blockers may be associated with an increased risk of depressive disorders, compared to other antihypertensive medication, which is consistent with our findings [42]. Altogether, the unexpected effects of specific medication require further clarification.

Our model suggests a significant association between hyperuricemia and an adverse outcome of depressive symptoms. It has been shown that elevated serum uric acid levels predict adverse outcomes after myocardial revascularisation or cardiac valve surgery [43]. Some authors maintain that the purinergic system is involved in the regulation of mood [44, 45], but current data regarding the relationship between serum uric acid levels and depression is inconsistent. Studies discovered a negative correlation between depression scores and serum uric acid levels [46–50], but one study found that hyperuricemia predispose to depression in patients with systolic heart failure, irrespective of the intake of uric acid lowering drugs [51]. In this context, it is important to note that depression and general discomfort are possible side effects of the drug “Allopurinol”, a common uric acid lowering drug. Similar to the discussion of thyroid medication above, a higher burden of disease, possible drug interactions and other unexplored factors might have influenced our findings.

Our finding of the unfavourable effect of higher triglycerides, a modifiable risk factor for CAD, on depression outcome may also be explained by a higher burden of somatic disease or a reduced health-related behaviour. It is interesting to note that the BMI or other parameters of metabolism and potential comorbidities had no influence on depression outcome. Another possible indicator of a higher burden of disease may be a previous resuscitation, which could also be shown in a study of 839 patients with heart failure, predicting minor or major depression after 12 months [52].

Surprisingly, an unknown previous myocardial infarction was associated with a favourable outcome after 18 months. Patients who could not remember whether they had a myocardial infarction prior to study enrolment may show a reduced feeling of sickness in general. However, this finding remains difficult to explain.

We found an association between greater heart rate variability and a favourable depression outcome, which is in line with previous findings where heart rate variability is discussed as a marker of emotional dysregulation and predicts an increased disease risk [53–55]. One study could show that a low (nighttime) heart rate variability was a significant predictor of a poor response to treatment of major depression in patients with stable CAD [56]. Caldwell et al. (2018) suggest the use of a heart rate variability biofeedback in combination with psychotherapy, improving the outcome of depression treatment [53]. As mentioned above, we could only show possible predictors of depression outcome in general and not of treatment efficacy, highlighting a lack of comparability of the cited literature and our findings.

Several limitations must be considered when evaluating these findings. As already described in the SPIRR-CAD randomised controlled trial, severely ill patients had to be excluded and many participants had recently experienced an acute cardiac event. Therefore, our data might not be generalisable to patients with chronic stable CAD. The number of participants taking thyroid or antibiotic medication is relatively small, but to our knowledge, the analysed dataset is from the largest European treatment trial for depressed patients with CAD and the second largest worldwide. Besides the information on specific medication intake, further measures e.g. for assessing thyroid metabolism, such as T3 or T4, were not available. We also have to consider possible drug interactions that cannot be detected in our study. Moreover, though our analyses did not indicate any significant interaction of variables with the (random) treatment assignment, the identified prognostic variables for depression outcome may still be candidates for (treatment) effect modification to be further investigated in future studies. In fact, the planned analysis of the SPIRR-CAD study already showed a significant treatment-by-Type D interaction on change in depressive symptoms [26]. In the present work we wanted to detect as many potential predictors of depression outcome as possible and we therefore chose an exploratory retrospective approach. As a consequence, we have to assume some coincidental results. Strengths of this study include the large sample size and the large spectrum of analysed variables. We screened each of the 141 variables carefully and considered possible interactions between our results, as well as the influence of comorbidities.

## Conclusion

The exploratory approach taken in this analysis provides information on possible predictors of depression outcome among patients with CAD. Literature concerning therapy response among this specific patient population is sparse. The evidence from the analysis should be verified in future research, analysing the unexpected influence of specific medication on depression outcome. If replicated in a future study, the consequences of the predictive information from this analysis for treatment decision-making should be considered.

## Additional files

Additional file 1: Screening variables of the SPIRR-CAD dataset. (DOCX 19 kb)

Additional file 2: **Table S2.** Predictive variables of depression outcome. (XLSX 15 kb)

## Abbreviations

ACE: Angiotensin-converting enzyme
ANOVA: Analysis of Variance
BMI: Body mass index
CABG: Coronary artery bypass graft surgery
CAD: Coronary artery disease
CCI: Charlson Comorbidity Index
CCS: Canadian Cardiovascular Society
EEG: Electroencephalography
HADS: Hospital Anxiety and Depression Scale
HADS-D: Hospital Anxiety and Depression Scale – German version
HRQoL: Health-Related Quality of Life
IQR: interquartile range
M: mean
MCS: Mental Component Summary
NYHA: New York Heart Association
PCI: Percutaneous coronary intervention
SCID: Structured Clinical Interview for DSM Disorders
SD: Standard deviation
SF – 36: Short Form Health Survey
SPIRR-CAD: Stepwise Psychotherapy Intervention for Reducing Risk in Coronary Artery Disease
T3: Triiodothyronine
T4: Tetraiodthyronine
TAU: Treatment as usual
TSH: Thyroid-stimulating hormone
