# Supplementary material for: Somatic and sociodemographic predictors of depression outcome among depressed patients with coronary artery disease - a secondary analysis of the SPIRR-CAD study
Source: BMC Psychiatry. 2019 Feb 4;19:57. doi: 10.1186/s12888-019-2026-6 (PMC6360727; doi:10.1186/s12888-019-2026-6)
Supplement: Supplementary file 1 — Screening variables of the SPIRR-CAD dataset. (DOCX 19 kb) [file 12888_2019_2026_MOESM1_ESM.docx]

**Screening variables of the SPIRR-CAD dataset**

sex (male/female)

age (years)

height (cm)

weight (kg)

reason for hospital admission (stable angina pectoris/unstable angina pectoris/NSTEMI/STEMI)

current treatment (conservative/interventional/coronary artery bypass/other)

severity of the event (light/moderate/severe)

coronary artery disease (yes/no)

previous myocardial infarction (yes/no/unknown)

number of myocardial infarctions

ischemic cardiomyopathy (yes/no)

previous heart failure (yes/no)

primary valvular heart disease (yes/no)

congenital heart disease (yes/no)

cardiomyopathy (yes/no)

bradycardic arrhythmia (yes/no)

tachycardic arrhythmia (yes/no)

previous coronary artery bypass surgery (yes/no)

number of coronary artery bypass surgeries

previous coronary intervention (yes/no)

number of coronary interventions

heart valve surgery (yes/no)

previous resuscitation (yes/no)

cardiac pacemaker (VVI/DDD/biventricular/no)

heart rate (beats per minute)

heart’s electrical axis (<-30°/-30 – 30°/30 – 60°/60 – 90°/90 – 120°/ >120°)

heart rhythm (sinus rhythm/atrial fibrillation/atrial flutter/pacemaker/other rhythm)

atrioventricular block (yes/no)

bundle branch block (yes/no)

heart hypertrophy (left/right/no)

ischemia signs (active ischemia/previous ischemia/previous infarction/none)

anterior/posterior myocardial infarction (anterior/posterior)

load limit stress ECG (watts)

ST-deviation (yes/no)

symptoms during stress ECG (yes/no)

left ventricular end diastolic diameter (mm)

left ventricular end systolic diameter (mm)

interventricular septal end diastolic diameter (mm)

posterior wall end diastolic diameter (mm)

left atrial end systolic diameter (mm)

ejection fraction (%)

valvular heart disease (yes/no)

valvular heart disease severity (I – IV)

coronary artery disease number of affected vessels

left main coronary artery (degree of stenosis)

left anterior descendens (degree of stenosis)

Ramus circumflexus (degree of stenosis)

right coronary artery (degree of stenosis)

coronary artery bypass graft (open/partly closed/closed)

CCS-class (0– IV)

NYHA-class (I – IV)

marital status (married and living together/married and not living together/single/divorced/widowed)

living together with a partner (yes/no)

living alone/living together (one person/more persons)

number of persons in household

educational qualification (none/“Hauptschule“/“Mittlere Reife“/“Polytechnische Oberschule“/“Fachhochschulreife“/“Hochschulreife“/other)

no educational qualification or not completed (yes/no)

professional training not completed yet (yes/no)

apprenticeship (yes/no)

school-based training/college/commercial school (yes/no)

technical college/master school/university of cooperative education/academy (yes/no)

university of applied science (yes/no)

university (yes/no)

other educational qualification (yes/no)

highest educational qualification (see above)

pension request (yes/no)

approved pension request (yes/no)

employed (>35 hours per week/15-35 hours per week/<15 hours per week/no)

reason for part-time work (disabled due to heart failure/disabled due to other reason/pensioner/unemployed/housewife or –husband/maternity leave/student/other)

monthly income

health insurance (statutory/private/social welfare office)

creatinine (level)

fibrinogen (level)

TSH (level)

cholesterin (level)

LDL (level)

HDL (level)

triglyceride (level)

hyperuricemia (yes/no/unknown)

blood pressure systolic (mmHg)

blood pressure diastolic (mmHg)

heart rate variability during dictated breathing rhythm (beats per minute)

heart rate variability during numeracy test (beats per minute)

heart rate variability during subsequent rest (beats per minute)

heart rate variability during dictated breathing rhythm sdnn (beats per minute)

heart rate variability during numeracy test sdnn (beats per minute)

heart rate variability during subsequent rest sdnn (beats per minute)

heart rate variability during dictated breathing rhythm rmssd (beats per minute)

heart rate variability during numeracy test rmssd (beats per minute)

heart rate variability during subsequent rest rmssd (beats per minute)

T0a: Hads-D-Depression-score

ACE inhibitors (yes/no)

aldosterone antagonists (yes/no)

analgesic drugs (yes/no)

antiallergic drugs (yes/no)

antianginal drugs (yes/no)

antiarrhythmic drugs (yes/no)

antibiotic drugs (yes/no)

anticholinergic drugs (yes/no)

antidepressant drugs (yes/no)

antihistaminic drugs (yes/no)

antipsychotic drugs (yes/no)

acetylsalicylic acid (yes/no)

angiotensin-II receptor antagonists (yes/no)

eye drops (yes/no)

beta blockers (yes/no)

beta-mimetic drugs (yes/no)

calcium channel antagonists (yes/no)

digitalis (yes/no)

diuretic drugs (yes/no)

uric acid lowering drugs (yes/no)

heparins (yes/no)

hormone preparations (yes/no)

hypnotic drugs (yes/no)

immunosuppressant drugs (yes/no)

inhaled corticosteroids (yes/no)

insulin (yes/no)

gastro-intestinal treatments (yes/no)

minerals (yes/no)

muscle relaxants (yes/no)

dietary supplements (yes/no)

neurologic drugs (yes/no)

NOAC (yes/no)

NSAID (yes/no)

opioids (yes/no)

oral antidiabetic drugs (yes/no)

phytotherapeutic drugs (yes/no)

thrombocyte aggregation inhibitors (yes/no)

thyroid substitution therapy (yes/no)

other antihypertensive drugs (yes/no)

other lipid lowering drugs (yes/no)

other medication (yes/no)

statins (yes/no)

systemic corticosteroids (yes/no)

thyreostatic drugs (yes/no)

tranquilizers (yes/no)

urologic drugs (yes/no)

virustatic drugs (yes/no)

coumarin derivate (yes/no)

vitamins (yes/no)

Charlson Comorbidity Index
